# How GPs communicate the urgent suspected cancer referral pathway to patients: a qualitative study of GP–patient consultations

**DOI:** 10.3399/BJGPO.2024.0115

**Published:** 2025-05-21

**Authors:** Jessica Russell, Laura Boswell, Athena Ip, Jenny Harris, Hardeep Singh, Ashley ND Meyer, Traber D Giardina, Afsana Bhuiya, Katriina L Whitaker, Georgia B Black

**Affiliations:** 1 Wolfson Institute of Population Health, Queen Mary University of London, London, UK; 2 School of Health Sciences, University of Surrey, Surrey, UK; 3 Center for Innovations in Quality, Effectiveness and Safety (IQuESt), Michael E DeBakey VA Medical Center, Texas, US; 4 Department of Medicine, Baylor College of Medicine, Texas, US; 5 Cancer GP lead for North Central London Cancer Alliance, London, UK

**Keywords:** Consultation skills, Diagnosis, Cancer, General practitioners, Primary healthcare

## Abstract

**Background:**

The UK National Institute for Health and Care (NICE) recommends that GPs inform patients referred onto the urgent suspected cancer (USC) pathway about what to expect from the service. However, there is a lack of evidence on patient experience and information needs at the point of referral. It is a challenge for GPs to communicate the reasons for referral and provide reassurance.

**Aim:**

To examine how GPs communicate a potential cancer diagnosis and USC referral in practice.

**Design & setting:**

This is a secondary analysis of a dataset of 23 audio-recorded GP–patient consultations, selected from a larger dataset of 200 consultations collected in Surrey and London, UK in 2017–2018. The consultations were selected based on inclusion criteria related to cancer discussions.

**Method:**

This is a qualitative analysis of video-recordings of face-to-face patient consultations.

**Results:**

We found that most GPs informed patients that they might have cancer and engaged in reassurance using personalised risk statements. Some GPs avoided all mention of cancer, using symptom-led language instead. GPs focused on communicating practical rather than support-based information. Although most GPs informed patients that they would be seen by a specialist within 2 weeks, few discussed patients’ support needs during the referral period.

**Conclusion:**

Clear communication about cancer in primary care is promoted in UK policy, and has an important role driving patient investigations attendance. The study highlights the need for further research on communication practices around cancer referral to improve patient understanding and experience. Our recommendations for enhanced communication may improve patient outcomes by optimising routes to diagnosis via primary care.

## How this fits in

Early cancer detection is a priority of the NHS. GPs refer patients with suspected cancer symptoms with the knowledge that the majority of patients will not be diagnosed with cancer. When communicating information to patients regarding the referral to cancer services, GPs must balance causing anxiety and losing patient trust with a potential delayed cancer diagnosis. National Institute for Health and Care (NICE) committee guidance has noted a paucity of evidence around patient information needs, particularly at the start of the cancer pathway.

## Introduction

In the UK, primary care patients with potential cancer symptoms are expedited through an urgent suspected cancer (USC) pathway, ensuring timely specialist consultation . Cancer investigation teams are incentivised to meet the Faster Diagnosis Standard to confirm or rule out cancer within 28 days.

Revised in 2015, the referral criteria now encompass symptoms with at least a 3% positive predictive value for cancer, significantly increasing the referral volume (by approximately 10% year on year).^1^ Despite GPs making an average of 65 urgent referrals annually, there is significant variation between GP practices in their use of different referral pathways, highlighting disparities in referral practices across demographics and GP practices.^2,3^

NICE recommends full disclosure to patients about their referral status and what to anticipate.^4^ Studies show that giving this information has both avoidant and engaging effects on patients’ attendance, presenting a significant challenge for GPs.^5^ Around 5–7% of patients with a symptomatic referral cancel or do not attend their hospital appointment, leading to worse early mortality outcomes for those with cancer.^5,6^ The NICE guidance committee noted a paucity of evidence in their last review (2022) around patient experiences and informational needs during the diagnostic pathway, particularly between primary care presentation and secondary care consultation.^7^ Our aim was to explore how GPs discuss the possibility of cancer and the urgent referral pathway to patients during primary care consultations, and to compare these discussions with NICE guidance. Our research questions included the following:

1. How is the possibility of cancer discussed during consultations with suspected cancer features, including the likelihood of the symptoms being cancerous?
2. How is the USC referral pathway explained to patients in primary care?
3. How do these discussions (in research questions 1 and 2) compare with NICE guidance (NG12) items 1.14.3 and 1.14.5?^4^

## Method

### Study design and public patient involvement (PPI)

Study design was developed with an advisory committee, consisting of clinicians, public representatives, and academics, as well as partner organisations and two focus groups with members of the public (*n* = 7 per group). Partner organisations were involved in meetings to agree on the focus of the research.

### Methodological approach

This is a secondary qualitative analysis of a dataset of video-recorded face-to-face GP–patient consultations collected in Surrey and London, UK, in 2017–2018. The recordings were collected from seven GP practices, with nine participating GPs. Full information on data collection, participant recruitment, and ethical approval for this dataset can be found in Amelung *et al*.^8^

### Sample

We derived a sample of 23 consultations from our total dataset (*n* = 200) for this study with the following inclusion criteria: ≥1 new presenting problem; persistent undiagnosed problem; cancer discussion or mention of the USC referral pathway; patients aged >50 years (see Table 1 and Appendix 1 for patient characteristics). All participants gave informed consent to have their consultation video-recorded at the time of the primary study being conducted.

**Table 1.** Patient and doctor sample characteristics

**Characteristic**	Patients (*n* = 23)	Doctors (*n* = 9)
**Mean age, years (minimum–maximum)**	67 (50–88)	47 (32–59)
**Gender,** ***n*** **(%)**		
Male	14 (61)	6 (67)
Female	9 (39)	3 (33)
Total	23	9
**Ethnic origin,** ***n*** **(%)**		
White/White British/White Other	21 (91)	8 (89)
Asian/Asian British	1 (4)	1 (11)
Black/African/Caribbean/Black British	1 (4)	0
Other ethnic group	0	0
**Highest education qualification, *n* (%)**		
Degree or higher degree	5	n/a
Below degree level	11	n/a
No formal qualification	6	n/a
Information not provided	1	0
**Mean years since accreditation as a doctor (minimum–maximum)**	n/a	14 (2–30)
**Accredited doctor trainer,** ***n*** **(%)**	n/a	6 (67)

### Analysis

NVIVO (version 11) qualitative analysis software was used to support coding and analysis. Our focus on cancer-related discussions arose inductively from a broader analysis of how GPs manage diagnostic uncertainty.^9^ Transcripts were analysed by JR with support from GB to assess whether a cancer-relevant discussion had taken place, defined by either:

- using terms to directly or indirectly identify cancer, for example, ‘cancer’, names of specific cancers, euphemisms (such as ‘something nasty/sinister/going on’) or cancer descriptors (such as ‘something causing a blockage further up’); or
- a suggested USC referral, defined by either GP mention of a specialist in the next 2 weeks or any mention of the patient being available in the next 2 weeks.

Where there was uncertainty as to whether cancer was being discussed or a cancer referral was being made, GB and JR reviewed the full transcript, including the context of the presenting problem.

One researcher (JR) coded all the transcripts noting whether an USC referral had been made, including specific referral pathway. Inductive coding was carried out to analyse how the referral and the risk of cancer were described, with coding approach and emerging themes discussed regularly within the authorship team. Each video was analysed separately, rather than grouping according to which GP was present. Finally, codes were compared to NG12 guidance sections 1.14.3, 1.14.4, and 1.14.5 (relating to delivery of information on the USC pathway to patients). We developed recommendations for future service improvement and practice through discussion with the study’s clinical authors.

## Results

Illustrative quotes are reported alongside narrative descriptions of the findings.

### How are the possibility and likelihood of cancer discussed during cancer discussions?

#### The possibility of cancer

GPs mentioned the possibility that the presented symptoms could be cancer with two-thirds of patients, using either the word ‘cancer’, the name of a cancer (for example, ‘melanoma’), or cancer euphemisms, such as ‘something sinister’ and ‘something nasty’.

GPs introduced the possibility that the patient’s symptoms could be caused by cancer in a number of ways. Some GPs used a staged approach; they would start with ‘warning shots’, stating they needed to know the cause of the symptoms, followed by euphemisms for cancer, and finally using the word ‘cancer’:

*’ ... from the point of view could this be something else going on, could this be something nasty, like cancer … ‘* [GP48]

GPs would also use a ‘think-aloud approach’ as if the patient and GP were identifying potential diagnoses together:

*’ ... if we think it could be melanoma, if we do think it could we … we would have to refer you to what is called the 2 week rule.’* [GP7]

Other GPs would mention cancer within the context of referral guidelines or the name of the referral pathway, leaving patients to infer that their symptoms could indicate cancer:

*’That’s a bit strange, so the reason I’m looking in this book there is different guidelines about cancer or worrying symptoms …’* [GP64]

One GP treated cancer not as something to be identified, but to be ruled out:

*’We do want to be completely sure it’s not anything it’s not anything sinister in any way. So we are lucky in this area … we can refer you quite straightforwardly to the breast clinic.’* [GP59]

When cancer was mentioned to patients as a potential cause of their symptoms, GPs mostly mentioned other differential diagnoses.

#### The likelihood of cancer

Having mentioned the possibility of cancer, GPs immediately gave reassurance, emphasising the unlikelihood of a cancer diagnosis, including using physical gestures:

*’ … if it was a non-suspected cancer it would be here, but as it is a suspected cancer, and literally –* [indicates with thumb and index finger] *the estimated very small probability that it is cancer, it’ll be in* [name of clinic]*.*’ [GP7]

When discussing the likelihood of cancer, GPs invariably used personalised risk statements; that is, they gave the patient reassurance that they personally were at low risk, with only one instance of population-level risk statements being used:

*’ ... we refer people for suspected cancer and it’s even if there’s only about a 2% chance it might actually be cancer; we’d rather refer 98 and it’s normal than miss those two … ‘* [GP264]

Individual risk statements often explained to the patient that they were highly unlikely to have cancer, either without further explanation or by describing specific risk factors (for example, age or weight). In one case a patient was told that the referral was just a quicker way for them to be seen by a specialist.

### How is the urgent suspected cancer referral explained to patients?

GPs predominantly used two strategies to explain the USC referral to patients:

- balancing the anxiety of a cancer referral with a short waiting time; and
- using symptom-driven language: no mention of potential differential diagnoses.

#### Balancing the anxiety of a cancer referral with a short waiting time

GPs who used this strategy typically explained the need to either establish a cause for worrying symptoms or ‘rule out’ cancer. They then acknowledged that they were adding cancer anxiety to diagnostic uncertainty, before focusing on the relatively short interval within which the patient would be seen by a specialist:

GP: *’We need to know a cause … could it be a nasty cause, that’s why they’ve done the tests, and that’s why the scan is being done … ‘*

P: *’That will just have me worried for a week.’*

GP: *’Well … hopefully the worry can at least be helped by the fact that actually they will move quickly and they should do that scan.’* [GP264]

#### Using symptom-driven language: no mention of potential differential diagnoses

In four consultations, GPs omitted any mention of the referral pathway or any potential differential diagnoses and instead focused on the need for these symptoms to be ‘checked out’ by a specialist:

GP: *’And what I’d suggest I’d do is if I write down to* [name of clinic]*, get the bowels checked out because obviously they know you pretty well, don’t they?’*

P: *’They do.’*

GP: *’The alternative is to go to* [name of hospital] *... but … it’s better to be in the same place … They should contact you within a couple of weeks.’* [GP255]

### How do GP communication strategies compare with NICE NG12 guidelines patient information and support 1.14.3 and 1.14.5?

GP communication strategies in comparison with the NG12 guidelines are shown in Table 2.

**Table 2.** GP communication strategies in comparison with the NICE NG12 guidelines

NG12 guidance 1.14 patient information and support	GP communication strategies
1.14.3 Explain to people who are being referred with suspected cancer that they are being referred to a cancer service. Reassure them, as appropriate, that most people referred will not have a diagnosis of cancer, and discuss alternative diagnoses with them [2015]	In *n* = 8 consultations, GPs told their patient that they were referring the patient to a cancer service. In *n* = 4 consultations, no mention of a cancer service was made. In the 8 consultations where GPs did inform their patient that they were referring them to a cancer service, GPs used reassurance. In *n* = 6 consultations, GPs offered patients both cancer and alternative diagnoses (four of which were initiated by the GP and two by patients). In *n* = 2 consultations, the GP mentioned only the cancer diagnosis, in *n* = 3 consultations there was symptom-directed language only and in *n* = 1 consultations only the alternative diagnosis was mentioned.
1.14.5 The information given to people with suspected cancer and their families and/or carers should cover, among other issues: • where the person is being referred to • how long they will have to wait for the appointment • how to obtain further information about the type of cancer suspected or help before the specialist appointment • what to expect from the service the person will be attending • what type of tests may be carried out, and what will happen during diagnostic procedures • how long it will take to get a diagnosis or test results • whether they can take someone with them to the appointment • who to contact if they do not receive confirmation of an appointment • other sources of support [2015]	In this dataset, in only one patient’s case did the GP not mention that the patient would be seen by a specialist within 2 weeks. In seven patients’ cases GPs informed patients where they would be seen (that is, the name of the hospital or clinic) In four patients’ cases, GPs spoke about the type of tests that might be carried out. No GP spoke about sources of support for the patient while they were waiting for the referral, whether the patient could take anyone with them, how to obtain further information and help and what to expect from the service

NICE = National Institute for Health and Care Excellence.

Consistent with these guidelines, most GPs informed patients that they would be seen specialist within a 2-week period. Over half of GPs also informed patients of the name of the hospital or clinic where they would be seen. Some GPs mentioned potential tests that might be carried out. We found zero instances where GPs discussed the patients’ support needs either while waiting or during their specialist appointment.

## Discussion

### Summary

This qualitative study, using audio data of GP–patient consultations, reports on how GPs talk about cancer and USC referral in practice. We found that GPs mostly do inform their patients that they could have cancer and engage in reassurance about the low risk of cancer, using personalised risk statements. A minority of GPs avoid all mention of cancer, using symptom-led language instead. GPs focused on communicating practical over support-based information.

### Strengths and limitations

A key strength of our study is the use of verbatim audio data. This type of data allowed us to capture *in vivo* GP–patient encounters rather than relying on reported data. The inclusion of both clinical and multidisciplinary academic researchers within the study team enabled us to integrate different perspectives in analysis and clinical interpretation of the findings.

A limitation of our study is that the data were not collected with the aim of capturing urgent referral conversations. In this smaller subset analysis, we only included consultations where we could identify that patients were urgently referred on a suspected cancer pathway. This resulted in a sample that was not a diverse, purposive sample of patients or GPs. Further research is required to explore how the need for urgent cancer investigations for suspected cancer are communicated in the context of differences in language and culture. Some patients are referred directly to investigations before seeing a consultant (for example, X-rays for suspected lung cancer) and these consultations would not have been captured in this sample. Similarly, the actual numbers of instances of certain communicative practices should not be interpreted as an estimation of the prevalence in practice. Notwithstanding this limitation, the presence and nature of certain communicative practices in this sample is likely to indicate its use in other GPs and geographical contexts. This data was collected before the COVID-19 pandemic and as such all consultations were face-to-face; further research is needed for phone and video consultations that have additional challenges such as disruptions and lack of visual feedback cues^10,11^ as well as post consultation written information sent to patients.

### Comparison with existing literature

Previous studies have focused predominantly on GPs’ referral practices on and reviews of the USC pathway.^12–14^ Very little research has been dedicated to understanding how GPs communicate the possibility of cancer and the referral pathway to patients in primary care, compared with the wealth of literature relating to communicating a cancer diagnosis.^15,16^

Our predominant finding is that GPs engage in communicative strategies to reduce cancer-related anxiety for patients, such as reassurances about low risk or avoidance of cancer terms. This is unsurprising given the significant emphasis in medical training on the need to balance informing the patient about potential serious disease while minimising anxiety.^17,18^ Similarly, in a large qualitative study, patients reported that GPs avoided cancer-related terms and used symptom-directed language instead.^19^ Concordant with our findings, that study found variation regarding communication of a potential cancer. Previous research has highlighted the potential distress when patients infer that they are being investigated for cancer on arrival at an oncology service or receive a letter after their secondary care consultation confirming the presence or absence of cancer without any prior warning.^20^ The symptom-led language identified in the current study (for example, ‘checking out symptoms’) could also result in confusion; patients may interpret ‘check out’ as a process that will investigate a number of options, whereas an USC referral pathway may only investigate a possible cancer.^21^ Our study identified a minority of consultations where GPs used only the name of the pathway (that is, leaving the patient to infer the possibility of cancer), or avoided both cancer terms and the name of the pathway. Our findings also demonstrate that some GPs use evidence-based communication strategies such as a staged approach.^22^

Our study has shown that GPs tend to use individual risk rather than population-risk information as recommended in NG12 guidelines. It is the difference between a GP telling their patient that ‘most people with your symptoms will not receive a cancer diagnosis, but we don’t want to miss those who do’ (population-level risk) against ‘you are unlikely to have cancer’ (individual-level risk). Previous studies on the way healthcare professionals communicate risk have focused on the mode^23–26^ and patients’ preferences.^27^ However, these studies have not sufficiently addressed the potential issue of individual-to-population frame-shift error in cancer referral.^28^ The USC referral pathway and associated NICE guideline constitute a population-level strategy for secondary prevention of late-stage cancer diagnosis. Further research is required as high levels of distress have been reported by people who later receive a cancer diagnosis after repeated healthcare professional assurances that they do not or are very unlikely to have cancer.^29^

Although our dataset was not collected explicitly to measure concordance with N12 guidelines, our study has highlighted that some of the communication-related guidelines are better used than others. Regional quality improvement efforts have promoted adherence to communication guidelines by using prompts in referral forms, but this has not been formally evaluated. In the case of guidelines relating to communication, this has not been established quantitatively, although other studies have noted that guidelines are most likely to be followed when they involve a definitive diagnostic action.^30^

### Implications for research and practice

The focus of research efforts has been largely to improve cancer outcomes by diagnosing cancer earlier. Key to this goal is understanding the patient experience of the referral pathway, so that any structural, communication, or emotional barriers to attendance of appointments and investigations and adherence to advice can be examined. There are limited studies on non-attendance of USC appointments;^5,31^ further research is needed on the influence of communication practices within the USC pathway on patient behaviour and experience to inform NICE guidelines (1.14). This could include analysis of non-verbal as well as verbal communication, such as gestures, behaviour, and written information. We also recommend further research on how the probability of cancer is communicated by healthcare professionals and understood by patients.

We recognise that GPs communicate in a way that is tailored to individual patients’ needs with the goal of maintaining the relationship; this may not always be well represented by the NICE guidelines, particularly where the GP anticipates high levels of fear from the patient. Nevertheless, on the basis of our findings we outline recommendations for communicating to patients around USC referral in Table 3, which could be included in future iterations of the NICE guidance.

**Table 3.** Recommended language and suggested language to avoid based on verbatim examples from our dataset

Scenario	Recommended communication	Verbatim examples of recommended communication	Suggested language to avoid	Verbatim examples of language to avoid
GP decides patient is in need of a referral to the urgent suspected cancer referral pathway	Explain that the patient is being referred on the urgent suspected cancer pathway	*’… so I think we have to refer you as an urgent suspected cancer because you’ve got sort of more than a month’s history of difficulty swallowing …’ [GP204]*	Avoid using solely symptom-directed language	*’As far as this is concerned* [pointing to left temple]*, I’ll contact the skin people, they’ll be at* [name of hospital]*, I think they’ll see you … in the next couple of weeks, just to be absolutely certain what’s going ok.’ [GP284]*
GP wants to explain why they are making a referral on the urgent suspected cancer referral pathway	Explain why the urgent suspected cancer pathway is being used	’*I think it’s worth referring you for further tests ‘cause you’re losing the weight.’ [GP232]*	Avoid stating that the only reason the GP wants a specialist appointment is to check out symptoms	*’And what I’d suggest I’d do is if I write down to *[name of clinic]*, get the bowels checked out because obviously they know you pretty well, don’t they?’ [GP255]*
GP wants to explain the 2-week-wait as a way to assuage patient anxiety	Use the reassurance of being seen quickly by a specialist	*’… you’ll be seen within 2 weeks, so really quite quickly.’ [GP248]*	Avoid intimating that the only reason that they are referring the patient is to get a rapid specialist opinion	*’It goes to the suspected cancer clinic but it doesn’t mean that you got cancer, it’s just a quicker way of getting the test done.’ [GP232]*
GP wants to explain the likelihood of the symptoms being cancer	Explain that most people referred will not go on to receive a cancer diagnosis	*’ ... we refer people for suspected cancer and it’s even if there’s only about a 2% chance it might actually be cancer we’d rather refer 98 and it’s normal than miss those two … ‘ [GP264]*	Avoid specifying the individual’s risk unless using a risk tool	’*I think it’s very unlikely having examined you that there’s something worrying or sinister cancer-like going on’ [GP64]*

In conclusion, clear communication about cancer in primary care is promoted in UK policy, and has an important role in driving patient attendance for investigations. Video and audio data of consultations has shown that GPs use different communication practices, particularly to reduce the anxiety associated with a cancer referral. Our recommendations for enhanced communication may improve patient understanding and experience, and have potential to improve patient outcomes, by optimising routes to diagnosis via primary care.

## Data

Data are available on reasonable request.
